# Scoping review protocol to investigate the experience of intimate partner violence among Black women and children living in the United Kingdom and how domestic violence specialist organisations support them to thrive

**DOI:** 10.1371/journal.pone.0340084

**Published:** 2026-02-03

**Authors:** Cynthia Dawes, Claire Powell, Carol Rivas

**Affiliations:** 1 Social Research Institute, University College London, England, United Kingdom; 2 Population, Policy and Practice Department, University College London, England, United Kingdom; 3 Social Research Institute, University College London, England, United Kingdom; Ben-Gurion University of the Negev Faculty of Health Sciences, ISRAEL

## Abstract

**Background:**

This protocol focuses on male-perpetrated intimate partner violence and aims to explore how Black women and children are supported to thrive post-intimate partner violence. Although this form of violence affects women across all cultures, Black women remain significantly underrepresented in the existing literature and often face various barriers to disclosure and help-seeking, including patriarchal silencing, immigration status, language issues and unsupportive attitudes of staff. It remains unclear how they transition from surviving to thriving individuals. The scoping review addresses the question: *What are the lived experiences of intimate partner violence among Black women and children, and what factors shape their concept of thrivership?.* The scoping review explores the knowledge gap in understanding how Black women and children affected by systemic oppression at the intersection of race, immigration and gender, experience thriving after leaving their abusive relationship. It examines existing research to identify key factors that contribute to their thrivership in the UK context. This study protocol provides a detailed outline of the planned methodology for conducting the scoping review.

**Methods and analysis:**

The scoping review of qualitative evidence will be guided by the five steps of the framework proposed by Arksey and Malley, which include identifying the research question, identifying relevant studies, selecting studies, charting the data, collating, summarising and reporting the results and an additional consultation stage with stakeholders. The results from the scoping review will be presented using the PRISMA Extension for Scoping Reviews (PRISMA-ScR), and the data will be analysed using thematic analysis. The databases searched will be Ovid PsycINFO, Scopus, ASSIA and Web of Science.

**Ethics and dissemination:**

The anticipated results from the review will help generate new ideas for future studies and inform policy. The findings will be submitted for publication in relevant peer-reviewed journals and presented at conferences and to appropriate stakeholders. No ethics is required as this is a review without human participants being involved. This protocol has been registered on the Open Science Framework (OSF).

## Introduction

According to the World Health Organisation (WHO), Intimate Partner Violence (IPV) refers to*:*

*“behaviour by an intimate partner or ex-partner that causes physical, sexual, psychological harm, including aggression, sexual coercion, psychological abuse and controlling behaviours”* [1]*.*

This includes, but is not limited to, physical violence (hitting, slapping, beating and kicking), sexual violence (forced sexual intercourse and other forms of sexual coercion), emotional abuse (insults, constant humiliation, threats to harm, intimidation, threats to take away children), and controlling behaviours (isolating the victim away from friends and family restricting access to financial support, employment, medical care or education [2]. Globally, IPV committed by a male intimate partner, husband, or ex-husband remains the most widespread form of violence against women [1]. While IPV can significantly impact the short-, medium--, and long-term mental and physical well-being of women and children, it can also have serious economic and social consequences for societies and countries [1,3].

A global review of intersections of violence with other situations reports nuances concerning a higher prevalence of violence against children and women across specific social and historical contexts and countries [4]. For example, in the Global North, disabilities, fewer citizenship rights, broader immigration legislature, health challenges, and poverty may mean that Black and Minority Ethnic (BME) women and children are inappropriately represented as victims [5–9]. In England and Wales, data from the year ending March 2024 shows that an estimated 1.6 million (6.6 per cent) women aged 16 years and over experienced domestic violence in the last year, compared to 712,000 men (3.0 per cent) [10]. In addition, for the year ending March 2024, the data reported no significant differences between any domestic abuse estimates across different ethnic groups [10]. However, data from the previous year (year ending March 2023) reported a significantly higher proportion of domestic abuse experienced by mixed and White ethnic groups compared with Asian or Asian British groups. In the same way, “almost twice as many women in the white ethnic group experienced domestic abuse in the last year (6.0 per cent) compared with Black or Black British women (3.1 per cent) and Asian or Asian British women (3.0 per cent)” [11].

Within this context, evidence shows that IPV remains misrecorded and underreported among BME women in the United Kingdom, which could potentially influence reports on national estimates. Studies cite various forms of patriarchal silencing, i.e., within families and communities that reinforce women’s attitudes [12–14]. For instance, in Nigeria, the value of family (such as keeping family matters/issues within the family) can further prevent Nigerian women in the UK from disclosing their experience of IPV to outsiders [12,15]. Likewise, research on IPV and gender norms in some cultures and countries supports the claim by feminists to explain men using violence against women, particularly in marriage. Here, the feminist perspective views IPV in relationships as a result of patriarchy, where practices and societal structures enable men to exploit, dominate and oppress women.

Often, terms used to describe race and ethnicity, such as ‘Black and Minority Ethnic’ (BME) and ‘Black, Asian and Minority Ethnic’ (BAME), can be problematic and generally misunderstood [16]. In the UK, the term BME or BAME is mainly used to describe those from non-white backgrounds or minority groups who are subjected to racist practices of exclusion and marginalisation [17]. In addition, the term BME entails any ethnic grouping, including white ethnic minority groups (for example, Gypsy, Roma and Travellers), but excludes native white British groups [16,18]. In this context, the scoping review will use focus on and preferentially use the term ‘Black women’ as this recognises that individuals who identify with this term are minoritised due to the social processes of racialised domination and power and not just by their distinct statistical minorities [19]. However, it uses the terms used in the papers and studies where this is more appropriate.

Studies have investigated the disclosure and help-seeking behaviours of BME women who experience domestic violence; the findings highlight immigration status, language problems and unsupportive attitude of staff as obstacles to disclosure [15,20,21]. Hence, these barriers place power on the abuser, who uses these as a weapon to ensure that their victims are caught in a double-bind [22,23]. In this case, if the victim remains in the relationship, they experience abuse- If they attempt to access resources and services, they face severe negative repercussions from the abuser and, in some cases, barriers to services [15,21,24]. In addition, some studies suggest that BME women experience barriers to accessing services, mainly due to systemic racism and policy exclusion [25,26].

Other barriers may exist for Black women who require safety for themselves and their children. In some cases, women experience many lengthy periods of abuse before being able to separate from their partners or family. For BME women, access to services, the feeling of being safe and long-term recovery for them and their children remain important issues that need to be addressed [7,27]. Similarly, hostile and discriminatory policies further exacerbate disadvantages to migrant women and children who require justice, safety and support services. For example, the No Recourse to Public Funds (NRPF) under broader UK immigration legislation increases further barriers to Black immigrant women and children [5]. Those on the NRPF status are residents subject to immigration control, who do not yet have Indefinite Leave to Remain (ILR) in the UK and who are not part of the European Economic Area (Non-EEA) [28].

In the US, previous research has shown that migrant women and children often struggle with different stressors that impact their attitudes and responses towards intimate partner violence [29,30]. For example, women’s vulnerability to abuse can be exacerbated due to their lack of awareness of legal rights and the available system in the host country. In the UK, immigrant women with insecure or unstable immigration status face other forms of abuse and control, together with domestic abuse, for instance, deportation threat, now known as “immigration abuse” [5].

Racialised inequalities are not adjacent but integral to how the health system responds to migrant women with NRPF [31]. This is further described in the concept of intersectionality by Crenshaw, which suggests that Black women experience the world (IPV included) differently than white ethnic groups due to intersecting factors such as race, gender, immigration and systemic responses [32]. It is well-established that BME women with insecure immigration status in the UK who are victims/survivors of IPV are best served by ‘By and for’ services, as they provide advocacy and holistic support that centre on women’s distinct intersectional needs [33]. However, these specialist services often come with challenges as they are mainly small, limited in capacity and numbers, and encounter significant issues with funding [33].

Above all, the impact of IPV and other forms of abuse negatively affects the mental health and well-being of Black women and children. Research on survival theory [34] indicates that women who experience domestic abuse often recognise their strengths and develop creative coping strategies. However, they are mainly unsuccessful in receiving adequate help or support due to institutional failure and systemic oppression. Although the term survivor is dominant and mainly used in the field, there have been some critiques [35] which suggest that the term survivor may not embody a complete outcome for healing as the term fails to explore a much longer-term recovery from abuse but rather focusing more on immediate freedom [36]. In this context, the term ‘thrivership’ offers a solution to the issues as it suggests that if women are thriving, they are growing, flourishing or prospering- thriving surpasses the lack of problems by signifying good health and well-being [8].

Similarly, thriving can be defined as an ongoing positive process of continuous growth through interactions with the human environment, which results in physical, social and psychological development and resilience [37]. Here, the positive end of thriving, also known as self-actualisation [38], is where survivors of IPV reach their peak (the survivors/thriver do not only overcome their traumatic experience but reach their fullest potential). Within this continuum process, each person grows and develops differently and at different rates based on their ongoing self-development and interaction with their environment [37]. The key concepts that shape and sustain thriving include factors such as personal perception, motivations and available resources. In this context, the way a woman relates to and navigates the adversities of her social environment, including the quality of interpersonal relationships [8].

Previous research indicates that spirituality often influences the thriving stage; women report feeling healed, no longer defined by their trauma, and committed to caring for their physical, emotional, and spiritual well-being [39–41]. Another study identifies the key components of thriving as providing safety, sharing one’s story, and social response [41]. While this study offers a standardised approach to how women thrive after IPV, a one-size-fits-all method for working with women from different backgrounds may provide limited insights into how services or practitioners can effectively support Black women through the thrivership process.

Hence, research on how Black women and children progress from surviving to thriving after IPV can help contribute to service responses and policy change. By recognising the strength and resilience of Black women, IPV specialist organisations can develop more effective, culturally sensitive interventions that foster a thriving journey towards self-actualisation. Additionally, contributing to policy change entails using a thriving model by policymakers to advocate for a more comprehensive policy that addresses the unique needs of Black survivors.

### Rationale for the scoping review

Conducting research on how Black women and children thrive post-intimate partner violence is crucial due to the increasing international demand for data and studies focusing on IPV [42]. Hence, by acknowledging the significant risks and vulnerability that some women experience, particularly Black women and children who are traditionally excluded from research, studies in this field can contribute significantly to effective service responses and informed policy changes. Despite some progress in this field, there remain some notable limitations, mainly with a lack of qualitative research exploring how Black women and children move from surviving to thriving. In addition, while previous findings on this topic contribute to our understanding of how women may survive abuse, it does not provide further information on what happens after the abuse and how Black women and children thrive afterwards [41]. In the same way, there is limited literature on children’s actions during intimate partner violence [43]. Addressing these gaps is crucial as this enables interventions and policies that are equitable, inclusive and effective in supporting the thrivership and well-being of Black women and children.

### Aim and objective

The scoping review investigates how Black women and children thrive after experiencing intimate partner violence. It highlights and examines the gap in existing literature concerning how these women and children, who face systemic oppression at the intersection of race, immigration, and gender, manage to thrive post-IPV. By analysing how research is carried out in this area and what studies are available, the review aims to identify key factors that contribute to their thrivership in the UK context.

## Methods

The scoping review will be guided by the five steps developed according to the framework proposed by Arksey and O’Malley [44], which complies with the Joanna Briggs Institute (JBI) recommendations for elaborating a scoping review and Levac et al.’s [45] additional consultation recommendation stage: [1] identifying the research question, [2] identifying relevant studies, [3] selecting studies, [4] charting the data, [5] collating, summarising, and reporting the results and [6] consultation. The results of the scoping review will follow the Preferred Reporting Items for Systematic Reviews and Meta-Analysis Extension for Scoping Review (PRISMA-ScR) [46], and data will be analysed using thematic analysis [47].

### Stage 1: Identifying the review question

The main research objective is to examine existing literature on lived experiences, thriving factors and barriers to thrivership. The aim is to understand what IPV look like for Black women and children, what helped them not just survive but thrive and what barriers stand in their journey to thriving (moving from survival to thriving). Hence, the following questions will guide the review:

1. What are the lived experiences of IPV among Black women and children?
2. What key factors contribute to the concept of thrivership after IPV?
3. What barriers do Black women and children experience in attempting to access support for their wellbeing?

### Stage 2: Identifying relevant studies

The search strategy will consist of a comprehensive literature search of both published and difficult-to-locate unpublished (grey literature) work. To identify relevant studies, the following databases: OVID PsycINFO, ASSIA Applied Social Science Index and Abstract, Web of Science and Scopus will be searched. Grey literature will be searched from the WHO database and IPV specialist organisation archives: Respect, Imkaan, SafeLives, Refuge and Women’s Aid. Other sources will include a list of potential papers obtained during hand-searching and scanning reference lists. All search outputs will be exported to the Rayyan software to remove duplicates, for title and abstract screening, and full text screening. Zotero will be used for data management. Recording screening will be finished by the end of May 2025, data extraction by June, and results are expected by October 2025. The search strategy developed for the OVID PsycINFO database will also be adapted and used in other databases.

The PCC (Population, Concept, and Context) framework guides inclusion criteria and helps create a precise and meaningful topic for a scoping review [44,48].

The search criteria are outlined below using the PCC framework;

- Population: Black women with lived experiences of intimate partner violence (age: women over 18 with children or without children – and studies that include Black children’s lived experiences of IPV).
- Concept: Experience of intimate partner violence.
- Context: Qualitative research studies.

The search strategies for the scoping review will include relevant synonyms and Boolean operators such as “AND” and “OR.” The search strategy will not be limited in date and the studies included will be English.

### Eligibility criteria

For this review, Black women and children refer to studies in the UK that include ethnic minority studies of Black African women, Black Caribbean women, African Caribbean heritage women, Black women of mixed heritage, Black British women and children who have experienced or have been exposed to IPV. Studies will not be limited to those that solely study how Black women and children are supported to thrive or look after their wellbeing after abuse, in case this misses relevant data from wider studies. The data collected will include examining how women and children transition from victim-survivors to thrivers, such as individual perceptions, resources available, types of support received, support systems and interventions, positive outlook, resilience, coping strategies, and perspectives on the future.

The review will consider studies conducted in the UK, including those in England, Wales, Northern Ireland, and Scotland. The scoping review will focus on qualitative studies that gather detailed data, such as semi-structured interviews, focus groups, perceptions, views, and experiences. It will include methodologies like grounded theory, phenomenology, ethnography, action research, case studies, and feminist research. Qualitative data are chosen because they provide in-depth insights into areas where current knowledge is limited, as in this case. Additionally, qualitative systematic review studies focusing on UK papers that meet the inclusion criteria will be considered, provided they include studies on Black women’s experiences of IPV. Studies involving Black, Asian, and Minority Ethnic women will be included if Black women are part of the study populations, as mentioned above. Mixed-method studies that include relevant qualitative findings for the review questions will be included (only the qualitative components will be extracted and analysed in this context).

### Exclusion criteria

Studies will be excluded if they do not meet the inclusion criteria above. Also excluded are:

- Studies where no abstract is available or full-text articles cannot be obtained.
- Dissertation, conference proceedings and narrative studies describing a theory or framework without primary research findings.
- Studies that do not include the experiences of intimate partner violence and are not clear on how Black women and children are supported to thrive.
- Studies which focus on quantitative data only.

### Stage 3: Study selection

The search results will be exported into Rayyan software for data management, where duplicates will be removed. Next, the title and abstract screening will follow based on the inclusion and exclusion criteria. Two reviewers will double-screen 20 per cent of the titles and abstracts to ensure consistency, and any disagreements that may arise will be resolved through discussion. The remaining titles and abstracts will be single-screened. The exact process will be applied to full-text screening.

The result from the final selection will be detailed in the scoping review and presented in full using the PRISMA Flow Diagram [49].

### Stage 4: Charting the data

This stage includes charting or extracting key information gathered from the primary data being reviewed. The data extraction tools used in the review will be adopted from the JBI. The data extracted will include significant information, Such as:

1. Author
  1. Year of publication
  2. Origin/country of origin
  3. Aims/purpose
  4. Population and sample size within the source of evidence
  5. Methodology/methods/data analysis
  6. Outcomes details
  7. Key findings that relate to the scoping review

Since the data extraction process requires an iterative approach, the tools can be modified if necessary. Given the depth of the review questions and the various sources of evidence that can be incorporated during the data extraction process, some relevant data items may emerge. Additionally, the peer-reviewed studies included in the review will undergo a quality appraisal assessment to rate their quality using the Critical Appraisal Qualitative Skills Programme (CAQSP) tool (2024) developed by Oxford University [50].

### Stage 5: Collating, summarising, and reporting the results

A narrative summary of data will outline how the findings from data extraction and selection relate to the review’s objective and question. The data from the review will be analysed descriptively, and the results will be presented using tables and figures followed by an in-depth inductive analysis (thematic analysis). Using thematic analysis can help examine the text, identify how the data relates to the research question, and create a label (code) that describes the text [47,51]. In addition, several tentative codes will be developed and modified iteratively during data analysis to help identify related patterns, which enables the creation of categories and themes [52,53].

### Stage 6: Consultation

The scoping review will include a consultation component with stakeholders from domestic violence specialist organisations (for example, staff from “by and for” organisations, police, and staff from the Domestic Abuse Commissioner (DAC) for England and Wales). Preliminary findings from stage 5 will be discussed with stakeholders of the scoping review to identify and seek feedback on the implications of the findings. The consultation with the stakeholders can provide additional information or references regarding the scoping review and the interpretation of its findings.

## Discussion and conclusion

This protocol provides the scoping review framework, which includes Arksey and O’Malley’s [44] five stages and a consultation stage. To our knowledge, this is the first scoping review that would investigate the experiences of IPV among Black women and children living in the UK in relation to how IPV specialist organisations support them to thrive. Including stakeholders in the scoping review will add value to the review as this will help provide a further perspective on the reviewing process. A key strength of the review is that it can provide a thorough and transparent approach to mapping out studies (data retrieval, screening and analysis) related to the experiences of IPV among Black women and children residing in the UK and how they are supported to thrive. A further strength of the review will be identifying themes that enable reporting the findings in an accessible format. One limitation of this study is the lack of diversity in the population sample; however, this is justified as Black women and children are often underreported or underrepresented in academic studies. Hence, the study is valuable as it specifically addresses the experiences of intimate partner violence among Black women and children.

Above all, considering policy development, systemic failure, and social inequality, there is a need to understand better the experiences and need to thrive of Black women and children residing in the UK in relation to IPV and to what extent these women and children are supported to thrive by IPV specialist organisations. This scoping review will shed some light on their experiences through an intersectional lens. Thus, race, gender, immigration status and systemic failures can help explain how these different forms of marginalisation can shape Black women and children’s experiences of IPV. The findings from this review will aim to inform opportunities for thriving that address the needs of Black women and children survivors of intimate partner violence.

## Supporting information

S1 File Search strategy. (DOCX)

S2 File Checklist. (DOCX)
