# Supplementary material for: Scoping review protocol to investigate the experience of intimate partner violence among Black women and children living in the United Kingdom and how domestic violence specialist organisations support them to thrive
Source: PLoS One. 2026 Feb 3;21(2):e0340084. doi: 10.1371/journal.pone.0340084 (PMC12867250; doi:10.1371/journal.pone.0340084)
Supplement: S1 File — (DOCX) [file pone.0340084.s001.docx]

# Supporting Information

## Search strategy

### PsycINFO Ovid

1. exp domestic violence/ or exp intimate partner violence/ or exp battered females/ or exp family conflict/ or exp marital conflict/
2. (domestic violence or intimate partner violence or family violence or honour-based violence or honor-based violence or battered women or domestic abuse or gender-based violence).mp. [mp=title, abstract, heading word, table of contents, key concepts, original title, tests & measures, mesh word]
3. 1 or 2
4. ("BME" or "BAME" or "Afro-caribbean" or "afrocaribbean" or "marginalised" or "immigrant*" or "marginalized" or "Minorit*" or "Black" or "African*").mp. [mp=title, abstract, heading word, table of contents, key concepts, original title, tests & measures, mesh word]
5. black people/ or "racial and ethnic groups"/ or african cultural groups/ or minority groups/
6. 4 or 5
7. ("women" or "woman" or "mother*" or "child*" or "adolescent*" or "teen*" or "girl*" or "young adult*" or "victim*" or "Survivor*").mp. [mp=title, abstract, heading word, table of contents, key concepts, original title, tests & measures, mesh word]
8. human females/ or battered females/ or daughters/ or mothers/ or sisters/ or wives/
9. Survivors/
10. qualitative methods/ or content analysis/ or ethnography/ or focus group/ or grounded theory/ or interpretative phenomenological analysis/ or narrative analysis/ or semi-structured interview/ or thematic analysis/ or exp interviews/ or lived experience/ or exp observation methods/ or phenomenology/ or exp qualitative measures/
11. ("Qualitative" or "thematic analys*" or "grounded theory" or "IPA" or "interpretative phenomenological analys*" or "narrative analys*" or "semi-structured interview*" or "ethnograph*" or "focus group*").mp. [mp=title, abstract, heading word, table of contents, key concepts, original title, tests & measures, mesh word]
12. 10 or 11
13. 7 or 8 or 9
14. 3 and 6 and 12 and 13
